# Yolk removal generates hatching asynchrony in snake eggs

**DOI:** 10.1038/s41598-017-03355-y

**Published:** 2017-06-08

**Authors:** Fabien Aubret, Florent Bignon, Alix Bouffet-Halle, Gaëlle Blanvillain, Philippe J. R. Kok, Jérémie Souchet

**Affiliations:** 1 Station d’Ecologie Théorique et Expérimentale, Oula-Lab, CNRS, UMR 5321, 09200 Moulis, France; 2 Amphibian Evolution Lab, Biology Department, Vrije Universiteit Brussel, 2 Pleinlaan, B-1050 Brussels, Belgium

## Abstract

Hatching synchrony is wide-spread amongst egg-laying species and is thought to enhance offspring survival, notably by diluting predation risks. Turtle and snake eggs were shown to achieve synchronous hatching by altering development rates (where less advanced eggs may accelerate development) or by hatching prematurely (where underdeveloped embryos hatch concurrently with full-term embryos). In Natricine snakes, smaller eggs tend to slow down metabolism throughout incubation in order to hatch synchronously with larger eggs. To explore the underlying mechanism of this phenomenon we experimentally manipulated six clutches, where half of the eggs were reduced in mass by removing 7.2% of yolk, and half were used as the control. The former experienced higher heart rates throughout the incubation period, hatched earlier and produced smaller hatchlings than the latter. This study supports the idea that developmental rates are related to egg mass in snake eggs and demonstrates that the relationship can be influenced by removing yolk after egg-laying. The shift in heart rates however occurred in the opposite direction to expected, with higher heart rates in yolk-removed eggs resulting in earlier hatching rather than lower heart rates resulting in synchronous hatching, warranting further research on the topic.

## Introduction

Synchronised hatching, a form of environmentally cued hatching^1^ (ECH), is widespread amongst organisms; including invertebrates^2^, fishes^3^, amphibians^4–6^, crocodilians^7^, squamates (snakes and lizards)^8–10^, turtles^11, 12^ and birds^13, 14^. Synchronised hatching is thought to enhance offspring survival by diluting an individual’s risk of predation or by simply swamping predators upon emergence^15, 16^. However mechanisms that promote hatching synchrony are poorly understood^11^. In both avian and non**-**avian reptiles^12, 17–20^, communication avenues amongst embryos or between embryos and parents may involve chemical cues, vibrations (including heart beats), acoustic cues and hypoxia^11^.

Reptiles are being increasingly recognised as a suitable model to study the inner mechanisms of embryo to embryo communication, for both logistical and biological reasons. That is, most reptiles do not attend nests and rarely exhibit parental care after oviposition^21, 22^. Clutches are also often quite large, allowing for powerful experimental designs; and eggs are relatively easy to incubate in a laboratory. Finally, heart rates (a reliable estimate of metabolism^23, 24^) of developing embryos can be easily and inexpensively monitored (i.e. egg buddies®).

Embryo communication in reptiles was shown to promote hatching synchrony primarily via metabolic compensation between more and less advanced eggs (in turtles^12, 18, 25^; and snakes^26^) or between large and smaller eggs (in snakes^10^). Experimental evidence^10, 12, 18, 25, 26^ suggests that synchronous hatching may be generated via (i) the alteration of development rates (i.e. metabolic compensation where less advanced eggs may accelerate development or smaller eggs may slow down development), or (ii) premature hatching (underdeveloped embryos hatch concurrently with full-term embryos, with potential detrimental effects). More specifically, the heart rate of young snake embryos was shown to be correlated with egg mass^10^. Embryos from smaller eggs tended to experience lower heart rates throughout the incubation but recovered a normal heart rate post-birth^10^, suggesting an adaptive cause to this physiological behaviour: embryos from smaller eggs slowed down metabolism in order to hatch synchronously with larger eggs.

While there is now good evidence that embryos can alter development rates by registering each other’s heart beats and thus metabolic levels within a nest^10, 12, 18, 25, 26^, the physiological mechanisms underlying the alleged relationship between egg size (i.e. yolk volume) and heart rate within an individual egg and amongst neighbouring eggs remains unknown.

To explore the inner mechanisms of hatching synchrony and the relationship between egg mass and metabolic rate, we collected clutches of the water snake *Natrix maura*: in each clutch, half of the eggs were reduced in mass by removing 7.2% of yolk using a syringe (i.e. allometric engeneering^27^). We wished to test two alternative hypotheses:

1. The relationship between egg size and heart rate is set prior to egg laying, in which case heart rate trajectories of yolk-removed eggs will be relatively unaffected compared to their siblings, potentially generating hatching asynchrony as smaller eggs might develop too fast for their mass.
2. The relationship between egg size and heart rate is not set prior to egg laying, in which case we expect yolk-removed eggs to compensate for their smaller size by slowing down development and hatching synchronously with control siblings.

## Results

### Experimental design and egg mass variation throughout the incubation

**Figure 1:**
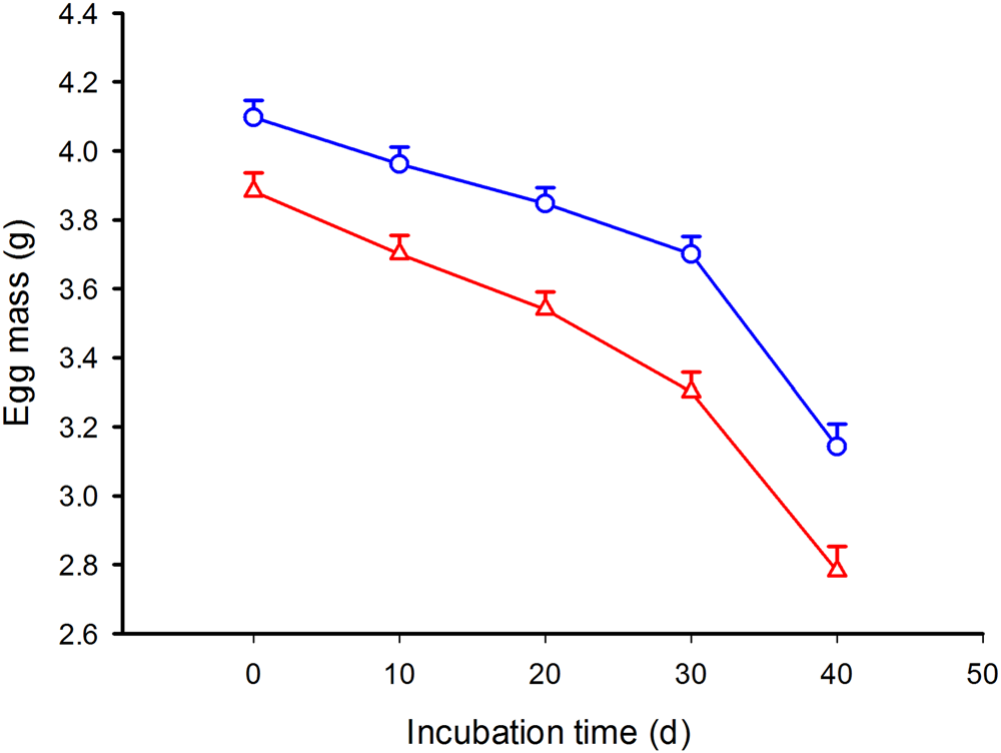
Egg mass trajectories were recorded every 10 days from laying to hatching in 30 water snake eggs from which 7.2% of yolk had been removed shortly after oviposition (red triangles) and 29 control eggs (blue circles). A repeated measure two-factor Anova with treatment and clutch of origin as factors and the successive egg masses as the repeated measure yielded: interaction term: F_5.34, 53.38 _ = 1.43, P = 0.22; effect of treatment over time F_1.78, 53.38_ = 1.99, P = 0.15; effect of treatment F_1, 30 _ = 24.05, P < 0.0001. As the assumption for sphericity was violated (W = 0.051, χ^2^_9_ = 84.33; P < 0.0001), results were corrected using Greenhouse-Geisser adjustments. Means ± SE are plotted.

The experimental design (split clutch design with mass-balanced egg allocation – see methods) generated comparable egg mass repartition between treatment and across clutches (two-factors Anova with treatment and clutch of origin as factor and initial egg mass as variable): interaction term F_4, 50_ = 0.25, P = 0.91; effect of treatment F_1, 50_ = 1.59, P = 0.22; effect of clutch of origin F_4, 50_ = 132.23, P < 0.0001. In the first group, some yolk was removed within 24 hours post-laying using a syringe (yolk-removed eggs; see methods) to generate a 7.21 ± 1.16% significant decrease in egg mass (from 3.98 ± 0.67 g to 3.68 ± 0.63 g; Wilcoxon Matched Pairs Test N = 30, T = 0.01, Z = 4.78, P < 0.0001). Eggs from the second group (control eggs) averaged 3.95 ± 0.65 g. This experimental design generated significantly different egg mass between yolk-removed and control eggs within each clutch (two-factor Anova with treatment and clutch of origin as factor and egg mass as variable): interaction term F_4, 50_ = 0.23, P = 0.92; effect of treatment F_1, 50_ = 16,17, P < 0.0002); effect of clutch of origin F_4, 50_ = 118,80, P <  =0.0001. This difference in egg mass remained significantly different, yet stable, throughout the incubation period between yolk-removed and control eggs (Fig. 1).

### Influence of yolk removal on heart rate – egg mass relationship

**Figure 2:**
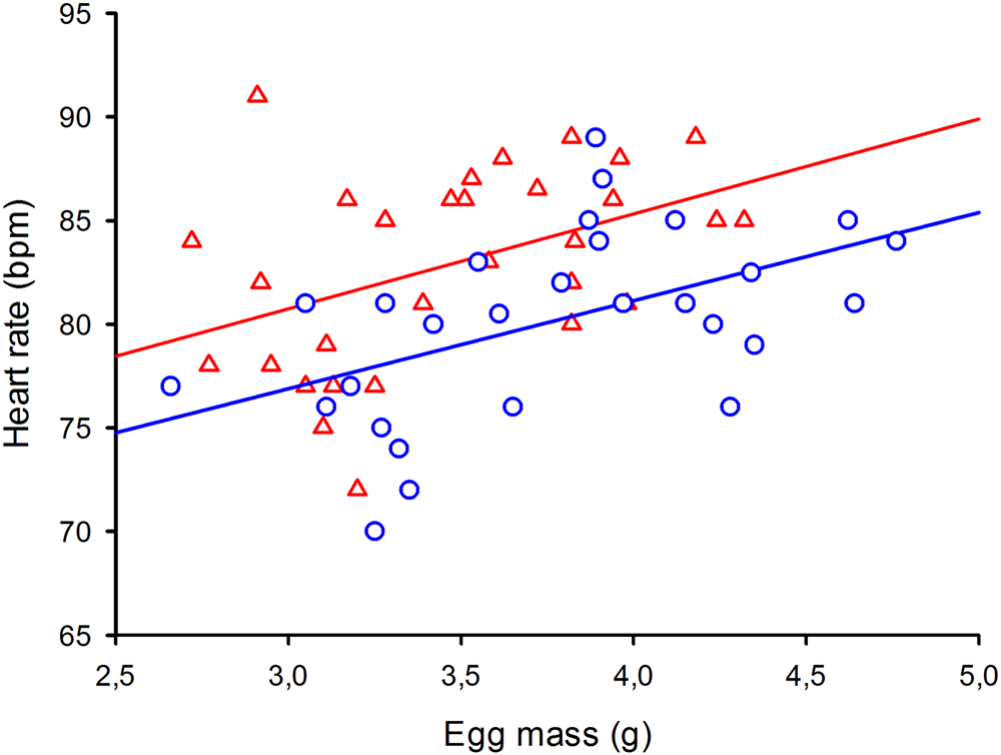
Early effects of yolk removal on the relationship between egg mass and embryo heart rates (incubation day 10) in yolk-removed (red triangles) and control water snake eggs. Heart rate and egg mass were strongly correlated in both treatments (see text for statistical details). While heart rate increased in response to yolk removal, the relationship between heart rate and egg mass was essentially unchanged. Means ± SE are plotted.

In order to describe the effect of yolk removal on the relationship between egg mass and embryo heart rates early in incubation (day 10), we ran a General Linear Mixed Model (GLMM) with heart rate as dependant variable, treatment (yolk-removed *versus* control eggs) as fixed factor, clutch of origin as random factor, and egg mass as continuous predictor (co-variate – Table 1). The relationship between heart rate and egg mass was essentially unchanged (Fig. 2). Heart rate and egg mass were strongly correlated in both treatments (linear regressions of heart rate against egg mass; yolk-removed eggs r = 0.72, F_1, 28_ = 29.31, P < 0.001; control eggs r = 0.47; F_1, 26_ = 7.22, P < 0.012). There was however a significant (and seemingly mechanical) increase in heart rate due to yolk removal in yolk-removed eggs compared to control eggs (Fig. 2).

**Table 1:** Statistical results of a General Linear Mixed Model (GLMM) with heart rate as dependant variable, treatment (yolk-removed *versus* control eggs) as fixed factor, clutch of origin as random factor, and egg mass as continuous predictor.

	Dl; F	P
{1} Egg mass	1; 0.39	0.54
{2} Treatment	1; 12.12	0.015
{3} Clutch of origin	4; 9.88	0.009
{2} * {3}	4; 1.20	0.32

**Figure 3:**
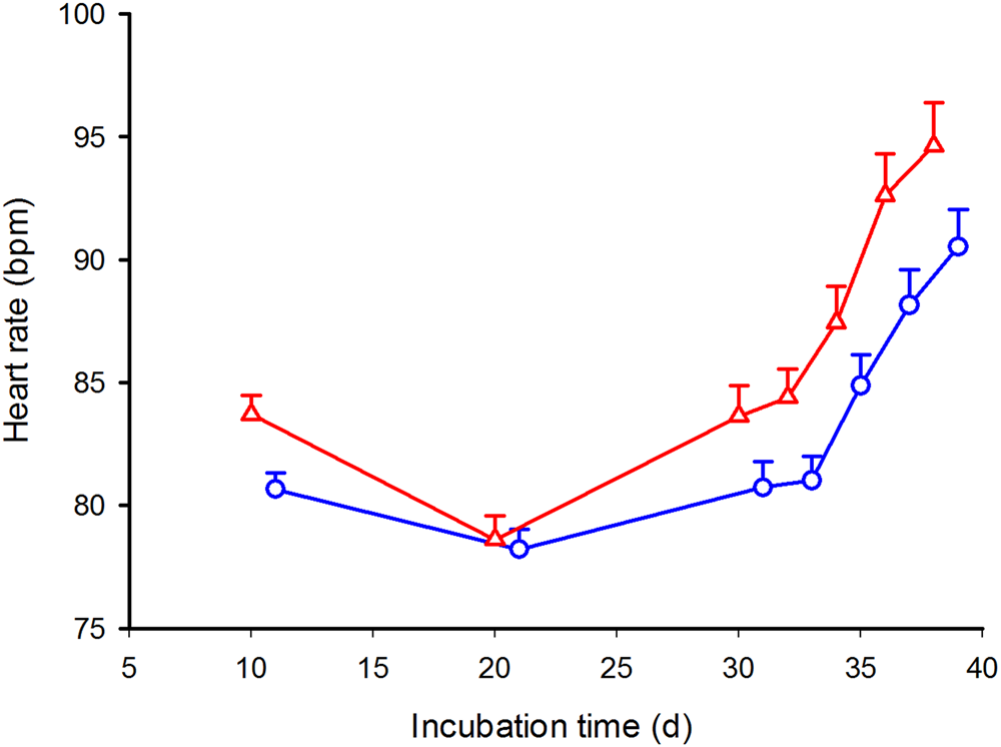
Heart rate trajectories were recorded from laying to hatching in yolk-removed (red triangles) and control water snake eggs (blue circles). A repeated measure two-factor Anova with clutch of origin and treatment as factors and 7 consecutive heart rate recordings as the repeated measure yielded: global interaction term F_12.92, 137.81_ = 1.32, P = 0.21; effect of heart rates over time interaction; F_4.31, 1137.81_ = 0.66, P = 0.63; effect of treatment F_1, 32_ = 10.48, P < 0.0028. As the assumption for sphericity was violated (W = 0.026, χ^2^_20_ = 39.67; P < 0.0001), results were corrected using Greenhouse-Geisser adjustments. Means ± SE are plotted.

Although heart rate trajectories were comparable between treatments, heart rates of embryos from yolk-removed eggs were consistently higher than heart rates from control eggs throughout the incubation period (see Fig. 3).

### Hatching success and hatchling phenotype

There was no significant difference between yolk-removed and control eggs in the proportion of eggs that successfully hatched (20 out of 30 *versus* 24 out of 30 respectively; Pearson χ^2^ = 1.36; df = 1; P = 0.24) and in the proportions of death post-hatching (3 out of 20 *versus* 4 out of 24 respectively; Pearson χ^2^ = 0.023; df = 1; P = 0.88).

Trait and performance comparisons and statistical results between yolk-removed and control eggs are presented in Table 2. Yolk removal significantly shortened incubation time and produced smaller hatchlings, both for body mass and snout-vent length. While yolk-removed eggs displayed higher metabolic levels throughout the incubation period (Fig. 3), heart rates at 3 weeks of age were lower (although not significantly) in snakes from yolk-removed eggs (Table 2).

**Table 2:** Two-way Anovas with treatment and clutch of origin as factors and relevant traits were performed. All interaction effects were non–significant therefore only data for main effect treatment is shown. Mean ± SE are given.

Traits	Intact eggs N = 21	Yolk removed eggs N = 20	df; F	P
Incubation time (days)	44.11 ± 0.27	43.21 ± 0.29	1, 33; 5.16	0.030
Unabsorbed yolk (g)	0.31 ± 0.040	0.33 ± 0.043	1, 33; 0.071	0.79
*At Birth*				
Body mass (g)	2.35 ± 0.067	2.04 ± 0.072	1, 33; 9.70	0.004
Snout-vent length (cm)	13.30 ± 0.19	12.46 ± 0.21	1, 33; 8.76	0.006
Body condition*	2.21 ± 0.044	2.15 ± 0.045	1, 35; 1.04	0.32
*Aged 21 days*				
Heart rate (bmp)	118.15 ± 1.64	113.30 ± 1.89	1, 27; 3.75	0.063
Body mass (g)	1.96 ± 0.049	1.85 ± 0.056	1, 27; 2.05	0.17
Snout-vent length (cm)	14.05 ± 0.17	13.73 ± 0.21	1, 27; 1.47	0.24
Defence score	3.11 ± 0.36	3.01 ± 0.414	1, 27; 0.0002	0.99
Swimming speed (cm.s^−1^)	46.04 ± 1.99	45.26 ± 2.16	1, 25; 0.069	0.80
Swimming speed (BL.s^−1^)	2.62 ± 0.10	2.65 ± 0.11	1, 25; 0.064	0.80

*Body mass relative to snout-vent length.

A two-factors Ancova with treatment and clutch of origin as factors, body mass loss between birth and 21 days of age as variable, and body mass at birth as covariate showed that young snakes born from control eggs tended to lose relatively more mass than those from yolk-removed eggs (0.42 ± 0.17 g *versus* 0.42 ± 0.18 g respectively): interaction F_3, 27_ = 2.38; P = 0.091; effect of clutch of origin F_3, 3_ = 5.00; P = 0.11; effect of treatment F_1, 3.19_ = 6.56; P = 0.078.

Relative growth in snout-vent length during the first 3 weeks of life showed a similar tendency between the two groups of snakes: interaction term F_3, 27_ = 0.45; P = 0.72; effect of clutch of origin F_3, 27_ = 7.85; P < 0.0007; effect of treatment F_1, 27_ = 3.91, P < 0.059. Snakes from yolk-removed eggs growth on average by 0.95 ± 0.65 cm *versus* 0.66 ± 0.52 cm in snakes from control eggs.

Finally, swimming performances and defensive behaviour were unaffected by the treatment (Table 2).

## Discussion

In this study we aimed to clarify some of the physiological mechanisms underlying the relationship between egg size and developmental rates in snake eggs, where smaller eggs tend to develop at a slower rate than larger eggs, fostering hatching synchrony. Our results do not support the hypotheses that (1) the relationship between egg size and heart rate is set prior to egg laying (in which case heart rate trajectories of yolk-removed eggs will be relatively unaffected compared to their siblings, potentially generating hatching asynchrony as smaller eggs might develop too fast for their mass); or alternatively (2) that the relationship is not set prior to egg laying (in which case we expect yolk-removed eggs to compensate for their smaller size by slowing down development and hatching synchronously with control siblings).

Developmental rates of yolk-removed eggs were clearly affected by our treatment, prompting rejection of hypothesis 1, but were not slowed down, as expected following hypothesis 2. That is, embryos from yolk-removed eggs displayed a lasting increase in heart rate compared to control sibling eggs (Fig. 3), generating hatching asynchrony: yolk removed eggs hatched almost 24 hours earlier than their control siblings.

Unsurprisingly, yolk-removed eggs yielded significant smaller young snakes (both in body mass and snout-vent length). Yet, at 3 weeks of age and without any food provided, differences in body size were no longer significant (incubation-induced effects on offspring are often transitory^28^). Young snakes born from yolk-removed eggs seemingly slowed down post-birth metabolism (heart rates at 3 weeks of age were lower than control snakes or lower than their own rates at birth?), which resulted in lesser body-mass loss compared to control snakes, without compromising growth in snout-vent length. While yolk removal affected metabolism and hatching body size, it did not compromise hatching success, hatchling survival nor locomotor performances and defensive behaviour.

This study further reinforces the compelling observations that developmental rates in reptilian eggs are influenced at multiple levels, both by abiotic^29^ (temperature, humidity) and biotic factors (embryo positioning, contact with older eggs, clutch size^10, 20, 26, 30^). While our results support the idea that developmental rates are related to egg mass (i.e. yolk content^10^) and that this relationship is not fixed prior to egg-laying, the shift in heart rates occurred in the opposite direction to expected, with higher heart rates in yolk-removed eggs resulting in earlier hatching rather than lower heart rates resulting in synchronous hatching. This shortening in incubation length may result from (i) a smaller yolk content to metabolise, (ii) higher metabolic levels, or (iii) a combination of both factors. Regardless, hatching asynchrony resulted. Because control eggs were also punctured by a syringe (sham procedure) it is unlikely that the observed increase in heart rates in yolk-removed eggs was related to an anti-parasite or anti-predatory response (environmentally cued hatching^11^) generating increased development rates. Yet, and given our current knowledge, it is the most parsimonious hypothesis to this (puzzling) result. Further research may help clarify this point by, for example, creating two yolk removal categories (with either a small or large amount of yolk removed) in order to try and decouple alleged anti-predatory/parasitism responses (constant) from egg size related metabolic responses (gradual).

## Material and Methods

### Experimental design

Gravid female *Natrix maura* were captured along the banks of the Lez River in south-west Ariège, France, in June and July 2014. A total of 60 eggs were obtained from 6 clutches between 14/07/2014 and 14/08/2014 (mean clutch size = 12.00 ± 4.42 eggs). Eggs were measured in mass to the nearest 0.01 g using a digital scale within 12 hours of oviposition. Eggs were individually marked for identification purposes with a pencil using a letter (coding for clutch of origin) and a number (egg number within each clutch). Because egg mass influences both embryo metabolism and hatching phenotype^10^, eggs were allocated to two treatments using a split-clutch design: eggs were ranked within each clutch from heaviest to lightest and evenly reunited into two half-clutches. This ensured consistency in egg mass across treatments (see results). Within each clutch, all the eggs from one half-clutch were subjected to yolk removal using sterile syringes (2 ml capacity) and needles (25 G; 16 mm × 0.5 mm). The embryo was located by sight and yolk removed on the opposite side to avoid potential damage. For the same reason, the syringe was only inserted a few millimetres inside the egg. Following puncture, betadine was applied to avoid infections to the egg and the hole covered with a small piece (3 × 3 mm) of sterile band-aid material that fell off within a few days. Eggs were then reweighed to calculate the amount of yolk removed. The same procedure was applied to the second half-clutch, with the exception that once the needle was inserted, no yolk was removed (i.e. sham procedure).

The two half-clutches were then re-united into a single clutch, randomly placed in a plastic container (20 cm × 15 cm × 5 cm) on a 2 cm layer of wet vermiculite and into an Aqualytic® incubation chamber set at a constant 28 °C.

### Heart rate measurements

We measured embryo and young snake heart rates using the Buddy® digital egg monitor (MK2, Avitronics) under the standardised protocol described for eggs^10^ and small reptiles and amphibians^24^ respectively. The Buddy® system works by shining an infrared beam onto the surface of the egg, detecting minute distortions caused by embryonic heart beats. The Buddy® monitor was left inside the incubator at all times to prevent temperature variation during heart rate readings. Each egg was gently placed onto the sensor pad for heart rate reading (a stable reading was obtained after approximately 30 seconds) and then returned to its clutch. Embryo heart rates were measured at incubation day 10, 20 and 30, and then every two days until hatching. Heart rates were also measured after birth in young snakes aged 21 days.

### Egg and hatchling measurements

Egg mass was measured every 10 days until hatching. Eggs were individually placed into small jewellery bags (5 × 4 cm, made of fine mesh material) a few days prior to hatching. This ensured juvenile snakes could be matched to their egg shell when multiple births occurred at the same time. Hatching occurred between the 29^th^ of August and the 28^th^ of September 2014. Hatchlings were measured in body mass (±0.01 g) and snout**-**vent length (±0.1 mm) within 12 hours of emergence. Siblings were housed together in plastic boxes (15 cm × 10 cm × 5 cm) with a water dish, shelter and paper towel as substrate.

All described tests below were performed on all hatchlings aged 3 weeks over three days using standardised procedures^31–34^.

### Swimming performance

A high-definition wide-angle digital camera (25 fps) was fitted above a linear 300 cm × 40 cm × 50 cm swimming track and used to record trials (recording section of 120 cm). The tank was filled with 10 cm of water adjusted to 25 °C using a reverse-cycle water chiller (TECO® TC15). The video was then edited on a computer and swimming speed calculated over the first five lengths of the track swum by each snake. The fastest performance was retained for each of the five lengths swum, and a fastest overall performance for the entire swimming test (the first length was usually the fastest).

### Defensive behaviour

Snakes were tested at room temperature (approximately 20 °C). Defensive behaviour ranged from (i) body positioning as an S-shape, (ii) flattening of the head and/or body, (iii) hissing, (iv) cobra-like posture (raising of the anterior body and head, ready to strike), to (v) striking at the threat (always with the mouth closed). Each behaviour equaled one point. Individual scoring thus ranged from 0 (no reaction) to 5 (full anti-predatory panel displayed).

All experimental protocols were approved by the Préfecture de l’Ariège, which provided capture, breeding, experimentation, release and ethics permits (Arrété #2012**-**11). All experiments were carried out in accordance with the approved guidelines. All females were returned to their exact site of capture within two weeks of egg**-**laying. Once tests were completed, young snakes were given their first meal (small dead minnows ranging from 0.5 g to 1 g; supplied by Armorvif®) prior to being released at the maternal capture site.

### Data analysis

Assumptions for normality of the data and equality of variances were tested on all variables (Lilliefors and Levene’s tests). Means ± standard deviations are given unless otherwise stated.
